# DPDQN-TER: An Improved Deep Reinforcement Learning Approach for Mobile Robot Path Planning in Dynamic Scenarios

**DOI:** 10.3390/s25216741

**Published:** 2025-11-04

**Authors:** Shuyuan Gao, Yang Xu, Xiaoxiao Guo, Chenchen Liu, Xiaobai Wang

**Affiliations:** 1 Mechanical and Rail Transit, Changzhou University, Changzhou 213164, China; s23050858050@smail.cczu.edu.cn (Y.X.); s24050858039@smail.cczu.edu.cn (X.W.); 2 Technology and Engineering Center for Space Utilization, Chinese Academy of Sciences, Beijing 100094, China; guoxiaoxiao@csu.ac.cn; 3 China Special Equipment Inspection and Research Institute, Beijing 100029, China; fengyun@csei.org.cn

**Keywords:** path planning, deep reinforcement learning, mobile robot, transformer, parameterized action space, experience replay mechanism

## Abstract

Efficient and stable path planning in dynamic and obstacle-dense environments, such as large-scale structure assembly measurement, is essential for improving the practicality and environmental adaptability of mobile robots in measurement and quality inspection tasks. However, traditional reinforcement learning methods often suffer from inefficient use of experience and limited capability to represent policy structures in complex dynamic scenarios. To overcome these limitations, this study proposes a method named DPDQN-TER that integrates Transformer-based sequence modeling with a multi-branch parameter policy network. The proposed method introduces a temporal-aware experience replay mechanism that employs multi-head self-attention to capture causal dependencies within state transition sequences. By dynamically weighting and sampling critical obstacle-avoidance experiences, this mechanism significantly improves learning efficiency and policy performance and stability in dynamic environments. Furthermore, a multi-branch parameter policy structure is designed to decouple continuous parameter generation tasks of different action categories into independent subnetworks, thereby reducing parameter interference and improving deployment-time efficiency. Extensive simulation experiments were conducted in both static and dynamic obstacle environments, as well as cross-environment validation. The results show that DPDQN-TER achieves higher success rates, shorter path lengths, and faster convergence compared with benchmark algorithms including Parameterized Deep Q-Network (PDQN), Multi-Pass Deep Q-Network (MPDQN), and PDQN-TER. Ablation studies further confirm that both the Transformer-enhanced replay mechanism and the multi-branch parameter policy network contribute significantly to these improvements. These findings demonstrate improved overall performance (e.g., success rate, path length, and convergence) and generalization capability of the proposed method, indicating its potential as a practical solution for autonomous navigation of mobile robots in complex industrial measurement scenarios.

## 1. Introduction

In the assembly and measurement of large-scale structures, mobile robots are increasingly becoming essential platforms for high-precision measurement and quality inspection tasks [1]. For instance, in the domains of aerospace assembly measurement, ship assembly, and heavy industrial equipment assembly, robots are required to execute tasks such as multi-view scanning and three-dimensional point cloud stitching on the surface of aircraft fuselage, hull shell, or large mechanical structures. These measurement tasks demand high levels of path continuity, scanning accuracy, and motion stability [2]. However, in real-world production environments, there exists a multitude of dynamic interference factors, including temporary tools, mobile operators, forklifts, and other dynamic obstacles. These factors necessitate time-critical obstacle avoidance and trajectory reconstruction capabilities while performing continuous measurement [3]. Conventional path planning methodologies, predicated on static maps or obstacle avoidance strategies that depend exclusively on collision detection, encounter challenges in meeting the dual criteria of low-latency operation and resilience in complex environments. Therefore, achieving high-precision and continuous path planning in dynamic, obstacle-rich environments has become a critical enabling technology for advancing the automation and intelligence of industrial measurement systems.

Conventional path planning methods mainly include graph-based search strategies and sampling-based approaches [4]. Graph-based methods, such as A* [5] and Dijkstra [6] algorithms, treat navigation as a graph search problem. They generate optimal paths by discretizing the environment and applying heuristic cost functions, and they can guarantee the discovery of a globally shortest path. However, their computational complexity increases significantly with map size, making them less effective in environments with dynamic obstacles. In contrast, sampling-based methods—such as Rapidly exploring Random Trees (RRTs) [7] and Probabilistic Roadmaps (PRMs) [8]—construct feasible paths by randomly sampling the configuration space, avoiding the need for precise environmental modeling. These methods are well-suited for high-dimensional spaces. Nonetheless, they typically suffer from limitations in convergence efficiency, path smoothness, and global optimality, particularly in dynamic and complex environments.

In recent years, Deep Reinforcement Learning (DRL) [9] has achieved remarkable breakthroughs in diverse fields such as autonomous driving, robotic control, and gameplay. By enabling agents to interact with their environments, DRL allows systems to adapt to dynamic and uncertain conditions by continuously updating their policies to maximize cumulative rewards. During training, DRL commonly incorporates an experience replay mechanism, which buffers the trajectories generated by interaction and randomly samples them to construct training samples [10]. This process helps to eliminate temporal correlations among samples and stabilizes policy updates. However, in dynamic environments, conventional experience replay mechanisms often disrupt the temporal structure of state sequences. This disruption can dilute or overlook critical states, such as obstacle-avoidance turning points and path reconstruction, which are essential for adapting to environmental changes. As a result, mobile robots may struggle to capture high-value experiences effectively, thereby reducing their responsiveness and adaptability. Furthermore, many navigation tasks require the simultaneous handling of high-level discrete decision-making and low-level continuous control to ensure path continuity. Traditional DRL approaches, which rely exclusively on either discrete or continuous action spaces, are inadequate for modeling such hybrid decision-making demands. To address this challenge, the Parameterized Action Space (PAS) [11] framework was proposed, with the Parameterized Deep Q-Network (PDQN) [12] being its most representative algorithm. PDQN integrates a Q-network for discrete action selection with a policy network for continuous parameter generation. This design provides significantly stronger representational capacity than traditional methods and has been widely adopted. However, PDQN still suffers from an important limitation: its parameter policy network employs a single shared architecture for all discrete actions and does not account for semantic differences between them. This shared structure is prone to gradient interference and semantic conflicts during training, which reduces the accuracy of continuous parameter generation. These issues are particularly pronounced in tasks characterized by strong environmental dynamics.

The Transformer model [13] has become a powerful tool in DRL due to its capability for temporal sequence modeling. Unlike traditional recurrent neural networks (RNNs) [14], it employs a self-attention mechanism that enables parallel processing of entire sequences and flexible modeling of long-range dependencies. This property allows Transformers to preserve causal structures in state–action–reward trajectories, thereby alleviating the loss of critical temporal information caused by conventional experience replay. Moreover, by capturing complex, nonlinear relationships among states, Transformers enhance policy adaptability in dynamic environments, providing an effective solution to the challenges of frequent obstacle avoidance and path reconstruction. 

In this paper, we propose an improved parametric reinforcement learning method called DPDQN-TER (Disentangled PDQN with Transformer-Enhanced Replay), which integrates a Transformer-based temporal modeling mechanism and a multi-branch parametric policy network to address the above challenges. The key innovations of this work are summarized as follows:

1. Transformer-based prioritized experience replay in dynamic environments: We redefine the experience replay mechanism as a sequence modeling task. By introducing the Transformer’s self-attention mechanism, our method encodes multi-level attention across interactive sequences in the replay buffer. This approach retains causal dependencies while ensuring sample independence, thereby enhancing the recognition and sampling efficiency of key states in dynamic environments. As a result, policy convergence is accelerated, and training stability is improved.
2. Multi-branch sub-network for PDQN: The original parametric policy network is decoupled into three sub-networks, each corresponding to a discrete action: “forward,” “left turn,” and "right turn." Each sub-network specializes in generating continuous parameters for its assigned action. This architecture allows flexible adjustment based on the semantic and dynamic characteristics of different actions, reduces computational overhead, and improves the deployment-time efficiency and accuracy of parameter generation.
3. Comprehensive experimental validation and performance evaluation: The proposed method is thoroughly evaluated in both static and dynamic obstacle scenarios and compared against several benchmark algorithms, including PDQN, Multi-Pass DQN(MPDQN) [15], and PER. Experimental results demonstrate that DPDQN-TER outperforms existing methods in success rate, convergence speed, policy stability, and generalization. In particular, it shows stronger performance and adaptability in dynamic environments, validating the effectiveness and practical relevance of the proposed mechanisms.

## 2. Related Work

### 2.1. Application of Deep Reinforcement Learning Network in Path Planning

DRL enables adaptive decision-making through end-to-end learning frameworks. Early research has primarily focused on discrete action space methods, such as the Deep Q-Network (DQN) [16] and its improved variants, including Double DQN [17] and Dueling DQN [18]. In comparison, continuous action space methods are commonly based on policy gradient algorithms. Representative examples include Deep Deterministic Policy Gradient (DDPG) [19], Proximal Policy Optimization (PPO) [20], and Soft Actor-Critic (SAC) [21]. These approaches are widely used for optimizing high-dimensional continuous control in mobile robot manipulation and navigation tasks. However, they often lack the flexibility to support hybrid control structures that involve both discrete and continuous actions. In addition, they tend to show poor generalization performance in dynamic environments. Consequently, their effectiveness in complex path planning scenarios is limited.

Therefore, Hausknecht and Stone [22] first introduced the concept of parameterized action space through the Parameterized Action DQN (PADQN), which combined Q-learning for discrete action selection with a parametric policy network for continuous control. Building on this idea, Xiong et al. [12] proposed the P-DQN (commonly referred to as PDQN), which further integrates the principles of DDPG and DQN. Their method allows direct optimization in the parameter space and significantly enhances training efficiency. Following this development, researchers proposed several improved algorithms to overcome the limitations of PDQN. For instance, Tavakoli et al. [23] introduced the Action-Parameterized Q-Network (APQN), which further enhanced the representation of hybrid action spaces and laid the groundwork for later structural optimizations.

To summarize, PDQN and its derivative architectures effectively represent mixed action spaces using a parameterized action space modeling strategy and are currently important tools for addressing complex path planning problems. However, their policy network structures often suffer from semantic coupling and parameter interference. Additionally, the traditional experience replay mechanism struggles to extract key state information in dynamic environments. Therefore, improving the expressiveness of policy representations and enhancing sample utilization efficiency remain core technical challenges for advancing path planning performance.

### 2.2. Research on Parametric Network Architecture

PDQN serves as a representative framework for addressing the challenges of mixed action spaces. It alleviates the curse of dimensionality that often affects conventional DRL methods by decoupling discrete action evaluation (Q-network) from continuous action generation (parametric policy network). Later studies have shifted attention to deployment challenges in dynamic, sparse-reward, or resource-constrained scenarios. For example, Xu et al. proposed an action-decoupled SAC approach to emphasize modularity in decision-making under complex control demands [24]. Feng et al. investigated parameterized reinforcement learning for UAV navigation under sparse reward conditions, revealing challenges in sample efficiency and reward signal design [25]. Yang et al. adapted PDQN for computation offloading in LEO satellite-edge networks, highlighting the trade-offs between policy quality and real-time responsiveness in resource-constrained environments [26]. These works collectively point to three key practical issues. First, when discrete actions share a common parameter generation network, the lack of semantic separation may hinder accurate parameter prediction. Second, shared parameterization can introduce mutual interference between action strategies during training, especially under imbalanced data distributions, leading to reduced learning efficiency and generalization. Third, the large-scale architecture required to accommodate all actions increases computational overhead, slowing inference and limiting the system’s ability to respond in real time—an issue particularly critical in tasks such as dense-obstacle navigation.

To address these issues, researchers have proposed various structural optimization methods in recent years. Zhao et al. proposed a novel architecture known as the Branching Parameterized Q-Network (BPQN) [23]. This architecture incorporates a division of the policy network into multiple sub-branches, with each sub-branch corresponding to a distinct discrete action. The novelty of this approach lies in the independence of the sub-networks, which collectively output continuous parameters corresponding to the specific actions. This approach enhances the specificity and action consistency of the policy; however, the scalability of the model is constrained in a high-dimensional parameter space. The Multi-Head Parameterized Actor-Critical (MH-PAC) [27], as proposed by Wang et al., incorporates a multi-head structure and an adversarial training mechanism, thereby enhancing the stability and robustness of the policy while preserving parameter decoupling. The primary advantage of this model lies in its superior generalizability, particularly in adapting to complex task-switching scenarios. However, it should be noted that the model structure is intricate, and the training process is highly sensitive to hyperparameter settings. Furthermore, Chen et al. introduced an action semantics embedding module into the Semantic-Aware Parameterized Policy Network (SAPPN) [28], thereby enabling the policy network to adaptively distinguish the control semantics of different action categories. This, in turn, enhances the generalization ability and action decision consistency in navigation tasks.

### 2.3. Review of the Mechanism of Experience Replay

The experience replay mechanism is a critical component for improving sample utilization in reinforcement learning. Conventional approaches enhance training stability by disrupting the temporal sequence of interaction data and performing random sampling. However, in dynamic path planning tasks, key states such as obstacle avoidance, turning points, and path reconstruction often exhibit strong temporal dependencies. Random sampling tends to destroy these causal structures, which prevents strategies from effectively identifying high-value experiences and weakens a model’s responsiveness to dynamic changes. To address this issue, Schaul et al. [29] proposed Prioritized Experience Replay (PER), which assigns priorities to experiences using the temporal-difference (TD) error, thereby accelerating the use of critical samples. Nevertheless, PER relies on local error signals, lacks global trajectory modeling capability, and performs poorly in terms of efficiency and stability in sparse-reward or dynamic environments. Chen et al. [30] later incorporated Hindsight Experience Replay (HER) [31] into the PDQN framework. This integration substantially improved convergence speed in sparse-reward scenarios and further verified the effectiveness of targeted experience-enhancement methods. Furthermore, Zhang and Sutton [32] observed that, although disrupting empirical data may improve training stability, it also reduces the policy network’s ability to model critical state-transition paths, particularly in partially observable environments (POMDPs). Overall, existing experience replay mechanisms mainly rely on heuristic rules such as TD-error to evaluate the importance of experiences. While they can improve sample utilization efficiency to some extent, two major limitations remain. First, the absence of sequence modeling makes it difficult to uncover latent causal relationships in experience, as reflected in state–action–reward trajectories. Second, manually designed sampling rules lack task-awareness and struggle to adapt to variations in environmental dynamics and reward sparsity, leading to an inability to reliably identify the state sequences associated with key behavioral decisions under sparse rewards.

In recent years, the Transformer has become a prominent tool in reinforcement learning research due to its advanced sequence modeling capabilities. Methods such as Decision Transformer [33] and Trajectory Transformer [34] have demonstrated the effectiveness of modeling long-term dependencies in offline RL. Building on this, researchers have sought to enhance the temporal structure of experience replay by employing Transformers to improve the utilization efficiency of key states. For example, Shin et al. [35] proposed a Hierarchical Embedding Representation (HER) model based on a Transformer for offline reinforcement learning. By encoding historical trajectories and reconstructing temporal dependencies between states, this approach significantly improved the responsiveness of policies to key states. Nevertheless, offline training remains limited in meeting the efficiency and latency constraints of online path planning. To address this, Zeng et al. [36] embedded the Transformer into a policy network, enabling dynamic adjustment of action outputs based on historical state sequences. Their approach achieved smooth navigation in dense obstacle environments and reduced decision latency and improved online decision quality.. However, the model is still constrained by inference overhead when deployed on large-scale maps. Li et al. [37] further investigated hierarchical path semantics using multi-scale Transformer architectures. Their multi-head design enhanced policy generalization in complex scenarios. However, the structural complexity of these models introduced inference delays, which can compromise the efficiency of online deployment. Overall, the studies indicate that Transformer-based sequence modeling is valuable for trajectory-level temporal structure, yet it also reveals an inherent trade-off between generalization capability and deployment-time efficiency when the Transformer remains within the control loop, particularly on large maps or under highly dynamic tasks. In contrast to these settings, the present work addresses a parameterized action space (discrete actions coupled with continuous parameters) and confines the Transformer to the training stage for trajectory-level scoring in experience replay, excluding it from the deployment control loop. Consequently, our problem formulation and evaluation protocol differ from approaches based on Decision Transformer and Trajectory Transformer; we instead prioritize improved navigation performance and stability in dynamic environments through a semantically decoupled multi-branch policy combined with Transformer-enhanced replay.

## 3. Methodology

To achieve a more efficient and high-performance, stable navigation strategy in dynamic environments, this paper introduces DPDQN-TER, a closed-loop autonomous navigation method based on a DRL framework. First, the navigation task is formulated as a Markov Decision Process (MDP), which establishes the foundation for state, action, and reward definitions. Building upon this formulation, the proposed method integrates two key components: a multi-branch parametric network architecture and a Transformer-based experience enhancement mechanism. The former decouples continuous parameter generation across different discrete actions to mitigate interference and improve policy stability, while the latter leverages self-attention to capture temporal dependencies in trajectories and enhance the efficiency of experience utilization. Together, these two components form the core of the DPDQN-TER framework for high-performance, stable navigation in dynamic environments.

The autonomous driving task is formulated as a Markov Decision Process (MDP) [38], defined by the tuple {*S, A, R, γ*}, where *S* denotes the state space, *A* denotes the action space, *R* denotes the reward function, and *γ* denotes the discount factor, described as follows:

- State space(*S*): The state space includes various types of information, such as the position of the mobile robot, the position of the target point, LiDAR measurements, image features, and local path points. Through feature fusion, these elements form a state vector with a total dimension of 108. Notably, the “LiDAR” (independently and identically distributed, i.i.d., from a uniform distribution *U* [0.5, 5.0] and “camera” (i.i.d. from a normal distribution N(0,1) channels are synthetically generated and environment-agnostic, serving as abstract placeholders without involving ray casting or actual image rendering.
- Action space(*A*): The method adopts a parameterized action design that combines discrete actions with continuous parameters. The discrete actions are defined as forward, left turn, and right turn, while the continuous parameters are defined as v∈[0, 2] m/s and $\omega \in [-\frac{\pi }{4},\;\frac{\pi }{4}]\;\mathrm{rad}/s$.
- Reward(*R*): The reward function encourages the robot to move closer to the target, avoid obstacles, and prevent collisions. It also penalizes deviations from the designated path. The objective is to jointly optimize stability and safety.
- Discount factor(*γ*): Strategy optimization considers both immediate and long-term rewards, ensuring that navigation strategies balance short-term responsiveness with global optimality.

**Figure 1:**
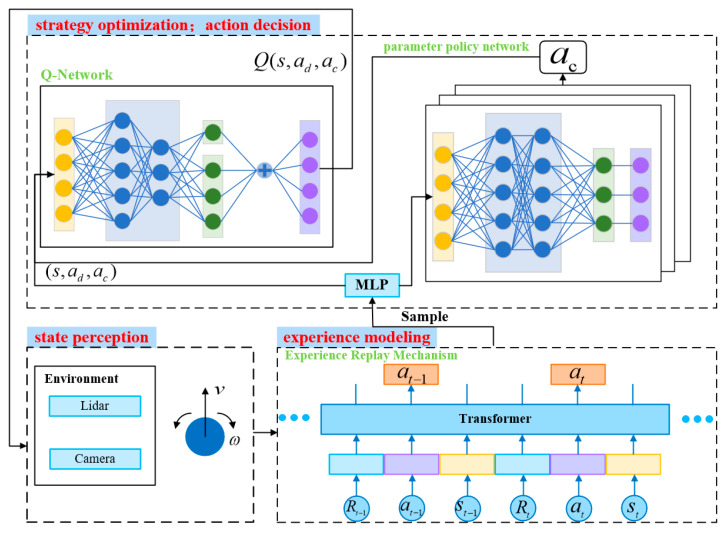
Autonomous navigation system framework for DPDQN-TER approach.

The overall architecture of the navigation system is illustrated in Figure 1. It consists of four stages: state perception, action decision, experience modeling, and strategy optimization. During interaction with the environment, the mobile robot selects an action (*A*) and receives a reward (*R*) based on its current state (*S*). This process generates experience samples that are stored in the replay buffer. Subsequently, the robot processes trajectory samples using a Transformer-based experience enhancement mechanism. The enhanced experience is then passed to a shared state feature extractor (MLP), which produces a unified state representation. This representation is input into both a Q-network and a multi-branch parameter policy network, enabling joint action evaluation and continuous parameter generation. The selected actions interact with the environment to generate new experiences, which are added to the replay buffer. Through this closed-loop cycle, the mobile robot iteratively improves its policy until convergence to an optimal navigation strategy is achieved.

### 3.1. Improved Multi-Branch Parametric Network Architecture

**Figure 2:**
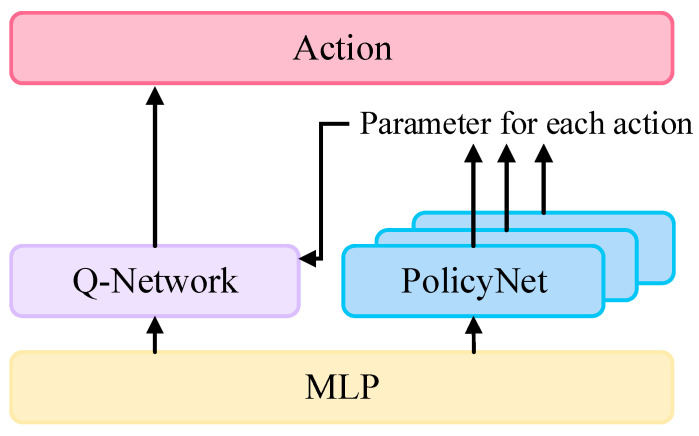
Multi-Branch Parametric Network Architecture in DPDQN-TER.

The improved multi-branch parametric network architecture of DPDQN-TER is illustrated in Figure 2. The core of the model consists of a shared feature extractor (MLP), a multi-branch parameter policy network, and a Q network. The MLP encodes the input state into a unified state feature vector Z, which is then shared by subsequent networks.

The parallel multi-branch structure contains three independent sub-networks, each responsible for generating continuous parameters for one of the discrete actions: forward, left turn, and right turn. Each sub-network is composed of three layers: an input layer, a hidden layer, and an output layer. The input layer is responsible for receiving the state feature vector Z, which is output by the multi-layer perception (MLP). The hidden layer is constituted by a fully connected layer comprising 64 neurons, employing the ReLU activation function to enhance the nonlinear expressive capability of features. The output layer’s design varies according to the action control objectives of the respective branches. The forward branch outputs continuous linear velocity v, which is constrained within $\left[0,\;v_{\max}\right]$ by a Sigmoid activation function; The left and right turning branches output continuous angular velocity ω, which is limited to $\left[-\omega_{\max},\;\omega_{\max}\right]$ by a Tanh activation function. In the kinematic model, the instantaneous motion of the agent is determined by the linear velocity v and the angular velocity ω. The sign of ω indicates the turning direction (left or right), while its magnitude reflects the turning rate. When ω≈0, the agent exhibits straight-line motion. Based on this property, the parameterized action space is decoupled into three discrete actions with distinct semantic meanings: forward, left turn, and right turn. This results in a minimal and non-redundant partition of the control manifold. This design offers several advantages. First, it aligns each network branch with a specific control behavior, thereby enhancing the interpretability of the learned policy. Second, by retaining v and ω as continuous variables instead of discretizing the turning magnitude, the approach preserves continuity within each branch and allows for fine-grained control. Third, it effectively reduces gradient interference that may arise when opposing turning behaviors are learned simultaneously within a single branch.

This design matches the bidirectional nature of rotational control, ensuring system effectiveness. By constraining parameter ranges, this configuration guarantees the physical plausibility of output parameters and improves training stability and convergence speed. Finally, the continuous parameters are combined with the discrete actions and passed into the Q-network, which evaluates the corresponding action values.

The Q-network is responsible for evaluating the value of actions $\left(a_{d}^{i},a_{c}^{i}\right)$. It adopts a fusion input structure comprising three components: The MLP outputs a state feature vector Z. The discrete actions are mapped into 8-dimensional embedding vectors, instead of using the traditional one-hot encoding. This embedding compresses the representation space and enhances the model’s ability to capture action semantics. The parameter input layer directly inputs continuous parameters corresponding to actions, such as linear velocity or angular velocity. These parameters are represented as real scalars, preserving their physical meanings. These three components are concatenated and fed into the backbone network, which provides a comprehensive state-action representation. The backbone is a three-layer feedforward neural network. The first layer is a fully connected layer with 128 units and a ReLU activation function, which extracts higher-order features. The second layer is a fully connected layer with 64 units, also using ReLU to increase nonlinearity. The final layer is a one-dimensional fully connected output layer that predicts the state-action value $Q(s,a_{d}^{i},a_{c}^{i})$ of the given combined action. The optimization objective is to minimize the mean-squared Bellman error:

(1) $$L_{TD}=E_{(s,a,r,s\prime )∼D}[{\left(y_{t}-Q(s,a)\right)}^{2}]$$

where $L_{TD}$ is the temporal difference loss function, D is the replay buffer, and $y_{t}$ is the target *Q* value, calculated as:

(2) $$y_{t}=r_{t}+\gamma \max_{a\prime }Q_{\mathrm{target}}(s_{t+1},a\prime )$$

where $r_{t}$ is the instant reward at time step t, γ is the discount factor, and $Q_{\mathrm{target}}$ is the target Q-network. This formulation allows consistent value comparison across parameter combinations of different discrete actions.

The sequence of actions executed by the mobile robot in a given environment is determined by the following process. At each interaction step, the robot receives the current state and generates continuous parameters for each discrete action independently through the multi-branch parameter policy network:

(3) $$x_{a_{d}^{i}}=\mu (s_{t},a_{d}^{i};\phi )$$

where μ(·;ϕ) denotes the policy network with parameter ϕ, and $a_{d}^{i}$ is the ith discrete action.

Subsequently, the Q network evaluates the state-action value for all possible combined actions $\left(a_{d}^{i},a_{c}^{i}\right)$, and selects the combination with the highest Q-value using an ϵ-greedy policy:

(4) $$\left(a_{d}^{*},a_{c}^{*})=\arg\underset{\left(a_{d}^{i},a_{c}^{i}\right)}{\max}Q(s_{t},a_{d}^{i},a_{c}^{i}\right)$$

where argmax is the maximization operator. After executing the selected action, the environment transitions to the next state $s_{t+1}$, and the immediate reward $r_{t}$ is received. This interaction experience $\left(s_{t},a_{d}^{*},a_{c}^{*},r_{t},s_{t+1}\right)$ is stored in the replay buffer for subsequent training.

### 3.2. Experience Enhancement Mechanism Based on Transformer

**Figure 3:**
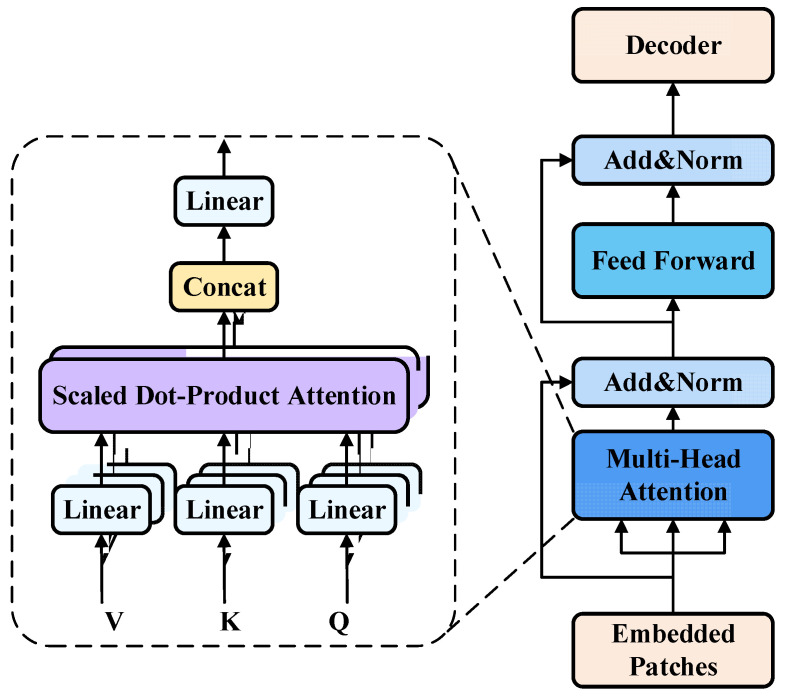
Structure flow of Transformer empirical encoder.

To achieve continuous policy optimization, the Q-network and the multi-branch parameter policy network are iteratively updated during training. Since their effectiveness relies heavily on the experience enhancement mechanism, this paper proposes a Transformer-based approach that reconstructs temporal correlations in experience trajectories through sequential modeling. It captures temporal causality within trajectories while maintaining sample independence. As shown in Figure 3, the proposed structure consists of two main components: an input embedding module and a multi-attention module.

In the embedding phase, the raw experiences collected during interaction are first organized into ordered trajectory data. Each trajectory is formalized as a multi-modal token sequence X, which is fed in parallel into a Transformer encoder. The state vector is projected into a fixed-dimensional feature vector using a linear layer. Discrete actions are mapped into dense vectors through an embedding lookup table. Continuous parameters are linearly projected and concatenated with the discrete action embedding to form a complete action vector representation. Rewards, as scalars, are also projected linearly to achieve a unified representation. In addition, position and type embeddings are incorporated to help the Transformer distinguish the time step and semantic category (state, action, or reward) of each token. Through these steps, the entire trajectory is represented as a unified input sequence to the Transformer.

In the encoding phase, the token sequence X is processed by a Transformer encoder composed of multiple layers, each containing a multi-head self-attention (MHA) mechanism and a feedforward network (FFN). The MHA performs global modeling of the trajectory sequence.

For each attention head, the query (Q), key (K), and value (V) vectors are constructed as:

(5) $$Q=XW^{Q},K=XW^{K},V=XW^{V}$$

The attention weights are computed as:

(6) $$\mathrm{Attention}(Q,K,V)=\mathrm{softmax}(\frac{QK^{T}}{\sqrt{d_{h}}})V$$

where Attention(Q,K,V) is the self-attention function, softmax is the activation function, $d_{h}=D/h$ is the single-head dimension, D is the embedding dimension, and h is the number of attention heads. The concatenated outputs of all heads are linearly transformed to obtain the multi-head attention output:

(7) $$\mathrm{MHA}(X)=[head_{1};\ldots ;head_{h}]W^{O}$$

where MHA(X) is the multi-head attention output, $head_{i}$ is the ith attention head output, and $W^{O}$ is the output weight matrix. This mechanism automatically extracts structural patterns from historical sequences, identifies critical states such as obstacle avoidance points, turning points, abrupt strategy changes, and path replanning events, and models their long-term dependencies with previous and subsequent experiences. The Transformer processes sequences in parallel and models arbitrary dependency structures, enabling comprehensive representation of high-value information.

In the Transformer output phase, for sequence-level scoring, the Transformer output sequence $Z_{t}$ is aggregated into a trajectory-level embedding via average pooling:

(8) $$h_{\tau }=\frac{1}{L}\sum_{t=1}^{L}Z_{t}$$

This embedding is fed into a feedforward scoring network that estimates the importance of each experience for the current policy. The scoring network is a two-layer fully connected network with ReLU activation, producing a scalar score that represents the learning value of each trajectory:

(9) $${\mathrm{score}}_{\tau }=W_{2}\cdot \mathrm{ReLU}(W_{1}h_{\tau }+b_{1})+b_{2}$$

where $b_{1},b_{2}$ are offset vectors of $W_{1},W_{2}$, respectively. For sampling, these scores are softmax-normalized to form an importance distribution:

(10) $$P_{\tau }=\frac{\exp({\mathrm{score}}_{\tau })}{\sum_{j=1}^{B}\exp({\mathrm{score}}_{\tau })}$$

where *B* is the number of trajectories in the sampled batch. During sampling, experiences are drawn from the replay buffer according to this distribution, with high-scoring experiences assigned greater probability. This allows the system to focus on high-value trajectories under limited training resources. Unlike conventional uniform replay or PER, which rely on local indicators such as TD-error, this method scores entire trajectories using global semantic information captured by the Transformer. As a result, it achieves stronger causal modeling and adaptability, making it particularly suitable for navigation tasks with sparse rewards or delayed feedback.

In summary, this section outlines the training mechanism and optimization process, which integrates trajectory modeling with a Transformer structure. The combination of experience scoring and weighted sampling based on semantic trajectory representation enables mobile robots to better leverage obstacle-avoidance capabilities and improve learning efficiency. At the network optimization level, the joint training of the Q-network and the multi-branch policy network further accelerates convergence and enhances policy stability. Overall, this mechanism provides a training framework with improved temporal perception and generalization for dynamic path planning. Building on this design, we briefly clarify its deployment-time complexity to facilitate a fair interpretation of the subsequent experiments. At deployment, the controller executes a shared backbone, the selected branch head, and a Q head; the Transformer module is used only during training for experience replay and is not part of the control loop. Consequently, the per-step inference complexity remains constant with respect to sequence length, and no online planning or search is involved. The additional computation at deployment is limited to a lightweight branch-head forward pass that is of the same order of magnitude as that of PDQN. This design confines sequence-modeling overhead to the training phase while preserving efficient and stable execution during online control.

## 4. Results

To comprehensively evaluate the performance and generalization of DPDQN-TER, a phased experimental design was adopted. First, basic tests were conducted in a static obstacle environment, which allowed for quantitative analysis of convergence efficiency and learning stability within the algorithm itself. These tests also verified the optimal performance level of the policy without external dynamic disturbances, providing a reference baseline for subsequent dynamic experiments. Second, experiments were conducted in a dynamic obstacle environment, which represents the core application scenario of this study. These tests examined the algorithm’s ability to handle on-the-fly obstacle avoidance in the presence of moving obstacles. In addition, ablation experiments were performed in Section 4.3 and Section 4.4 to analyze the independent contributions of the multi-branch structure and the Transformer-based experience mechanism. Finally, in Section 4.5, the generalization ability of the proposed method in non-customized scenarios was further validated through cross-environment testing.

### 4.1. Experimental Environment Setup and Evaluation Protocol

**Figure 4:**
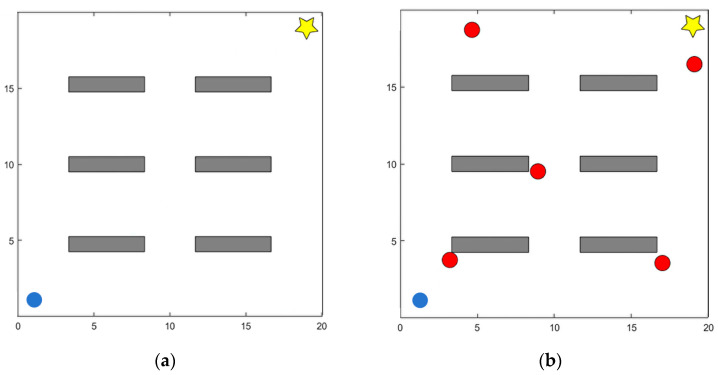
Simulation environment for evaluating DPDQN-TER: (**a**) Static Scenario; (**b**) Dynamic Scenario.

All experiments were conducted in simulated environments. Specifically, a two-dimensional continuous space simulation was used to model the navigation and obstacle avoidance tasks. Unlike grid-based methods that discretize the environment into cells, this approach employs a continuous coordinate system to accurately represent both positions and obstacles. Such a representation better reflects real-world navigation requirements and enables the simulation of more complex spatial dynamics. As shown in Figure 4a, the setup consists of a 20 × 20 static continuous map. The initial position of the mobile robot is marked by a blue circle, the target position is represented by a five-pointed star, and static obstacles are indicated by gray rectangles. As shown in Figure 4b, the dynamic environment is constructed by introducing five dynamic obstacles that move randomly within a specified velocity range v∈[0, 2] m/s, $\omega \in [-\frac{\pi }{4},\;\frac{\pi }{4}]\;\mathrm{rad}/s$. This modification simulates complex dynamic disturbances for testing purposes.

A series of experiments were conducted on a computer equipped with an NVIDIA RTX 4050 GPU, 64 GB of RAM, and an Intel Core i5-12490H CPU. The simulation environment and DRL policy algorithm were implemented in Python 3.8, with neural networks developed using TensorFlow. The configuration of the DRL algorithm is presented in Table 1.

The training process consisted of 1000 episodes, with a maximum of 200 steps per episode. To encourage the agent to explore the environment more thoroughly at the beginning of training, a fixed-step mechanism was applied during the first 100 episodes. In this phase, if the agent collided, went out of bounds, or reached the target point, it continued executing actions until 200 steps were completed. This ensured maximum coverage of the state space. After the 100th episode, the interaction rules were adjusted so that a collision, boundary crossing, or successful arrival at the target immediately terminated the episode. The cumulative reward obtained in each episode was recorded for subsequent training.

To ensure fair comparison, all algorithms are executed under strictly identical conditions. During training, a fixed random seed keeps the initial environment state (start/goal locations and obstacle layout) consistent across runs; during testing, a different set of seeds is used to generate initial positions and motion trajectories of dynamic obstacles, reducing overlap with training conditions and enabling a fair assessment of generalization and performance stability in unseen scenarios.

To evaluate the algorithm’s overall performance, the following metrics were defined and used in this study: The Average Cumulative Reward Value is indicative of the average level at which the strategy receives rewards over the course of training. The standard deviation is used to measure the degree of dispersion of multiple experimental results and evaluate the stability of the algorithm. The number of convergence rounds indicates the average number of training rounds required for the algorithm to achieve stable performance in the training phase, thereby reflecting the convergence speed of the algorithm. Success Rate represents the ratio of the number of times a mobile robot successfully reaches a target to the total number of tests, thus serving as a key indicator to measure the effectiveness of navigation strategies. The Average Path Length metric quantifies the mean length of a trajectory necessitated by a mobile robot to execute a task, thereby facilitating the assessment of path planning efficacy. Together, these metrics provide a comprehensive assessment of the success, efficiency, and stability of the strategy, enabling detailed comparisons among different algorithms in path planning tasks. The Minimum distance metric quantifies the closest distance between the planned path of the robot and any nearby obstacles during navigation. It reflects the safety margin maintained by the policy when maneuvering through dynamic environments. A larger minimum distance indicates a safer trajectory, while values close to zero may reflect risky behaviors or potential collisions.

**Figure 5:**
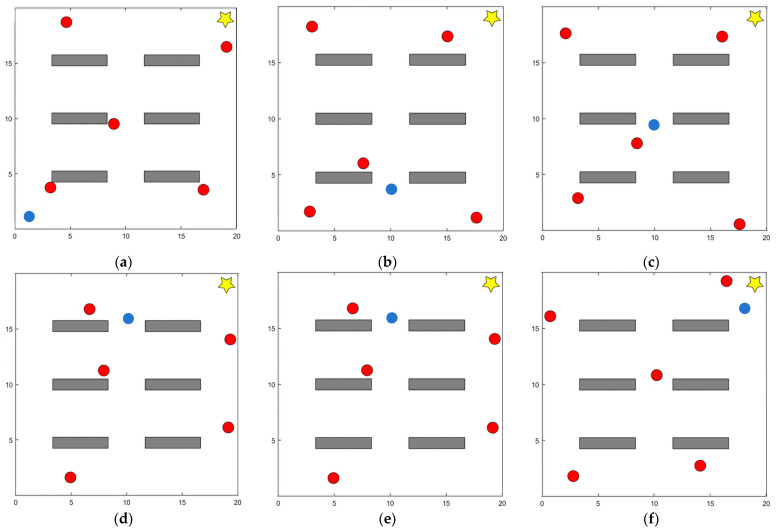
Bird’s-eye view snapshots of the robot’s path planning process in the dynamic scenario: (**a**) the robot starts from the initial position and detects the first moving obstacle ahead; (**b**) the robot executes an early avoidance maneuver, adjusting its heading to the left; (**c**) the robot completes the avoidance and reorients toward the target; (**d**) the robot encounters a second dynamic obstacle moving across its path; (**e**) the robot performs a right-turn avoidance and path reconstruction; (**f**) the robot successfully reaches the target while maintaining a smooth trajectory. These sequential snapshots illustrate how the proposed DPDQN-TER policy enables time-critical obstacle detection, avoidance, and path recovery in a dynamic environment.

**Figure 6:**
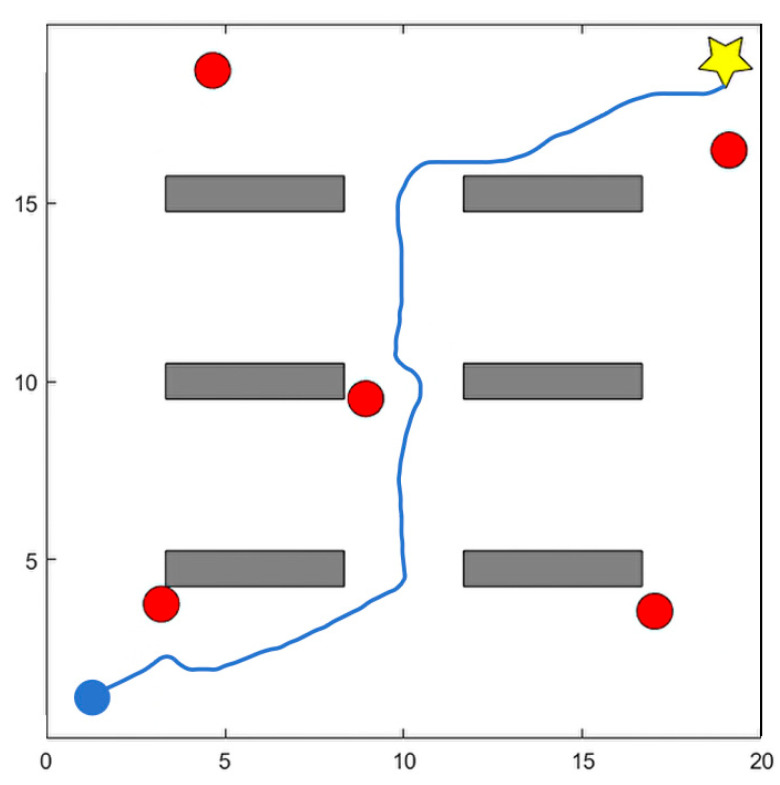
Visualization of the path planning process in autonomous navigation tasks.

Figure 5 presents six sequential snapshots from a representative dynamic-scenario test. Each subfigure corresponds to a key moment in the robot’s navigation process, including the initial motion, obstacle detection, avoidance maneuvers, and final target arrival. This visualization highlights how the DPDQN-TER policy dynamically adjusts its trajectory to handle multiple moving obstacles. This figure is generated from two-dimensional coordinate data in the simulation environment. Obstacles, starting points, target points, and robot trajectories are recorded in coordinate form and mapped onto a planar coordinate system using Matplotlib 3.5 to render the environment and trajectory layout from a top view. Figure 6 depicts the dynamic position changes of the robot during task execution, derived from the state sequences generated through interaction in the experiment. The system records the position and poses coordinates of the robot’s center point at each time step and connects them into continuous curves based on temporal order. This representation directly reflects the robot’s motion trajectory and obstacle-avoidance behavior in the dynamic environment. Since performance differences among algorithms are relatively minor in static environments, we visualize only the dynamic scenario here to more clearly highlight obstacle-avoidance behavior and path reconstruction. Together, the two figures highlight the complexity and dynamics of the experimental environment and provide intuitive support for the subsequent analysis of results.

### 4.2. Comparison of Training and Test Results

**Figure 7:**
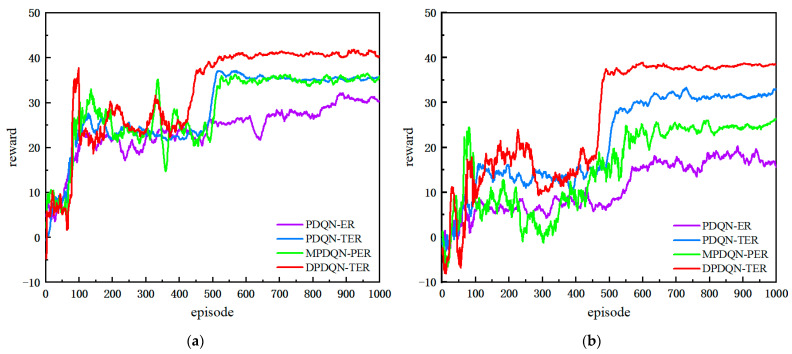
Comparison of Training Reward Curves Across Algorithms: (**a**) Static Scenario, (**b**) Dynamic Scenario.

In this section, we compare DPDQN-TER with three baselines: PDQN-ER, PDQN-TER, and MPDQN-PER, under both static and dynamic scenarios. We evaluate training behavior and test performance. Figure 7 presents the training reward curves for the two scenarios, and Table 2 summarizes the quantitative test metrics, including success rate, path length, and average reward.

As shown in Figure 7, the training reward curves are presented for both static and dynamic settings. In the static environment, all four methods complete the path-planning task. DPDQN-TER achieves higher success rate, shorter path length, and larger average reward than others, indicating better task efficiency and policy stability. In the dynamic environment, traditional methods exhibit lower success rates and larger convergence fluctuations as obstacles move. In contrast, DPDQN-TER maintains stable convergence and higher reward levels. Table 2 provides a quantitative comparison. In the dynamic scenario, the success rate of PDQN-ER drops to 0.60 and the average path length increases to 44.48, suggesting limited adaptability to environmental changes. MPDQN-PER improves slightly but remains sensitive to local anomalies. DPDQN-TER attains a success rate of 0.92 with a shorter path, demonstrating strong effectiveness and resilience in dynamic settings. In addition, the minimum distance to obstacles attains an intermediate level under DPDQN-TER, providing sufficient clearance while avoiding unnecessary detours; this balances safety with path-length efficiency in dynamic scenes.

**Figure 8:**
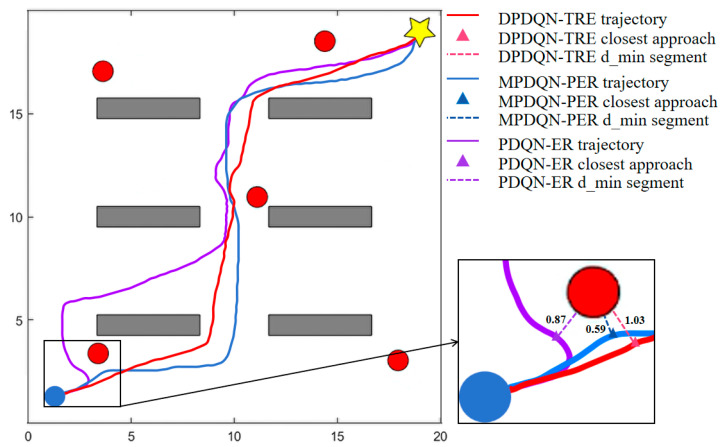
Trajectory Comparison of Different Algorithms in Dynamic Scenarios.

Figure 8 illustrates representative trajectories in the dynamic scenario. PDQN-ER produces long paths with many detours. MPDQN-PER reduces detours but remains unstable. DPDQN-TER yields smoother and more direct trajectories, enabling efficient obstacle avoidance and reliable goal reaching. These observations further support the superiority of the proposed method in dynamic scenes.

In summary, the comparative experiments demonstrate clear advantages for the proposed approach in both static and dynamic environments. Gains are mainly attributed to the multi-branch configuration, which reduces interference among actions, and the Transformer-based mechanism, which improves the utilization of key experiences.

### 4.3. Performance Analysis of Multi-Branching Strategy Structure

To verify the effectiveness of the improved multi-branch strategy, the proposed method (DPDQN) is compared with PDQN and MPDQN. Each method is trained five times in static and dynamic environments, with 1000 training episodes per environment. A uniform experience replay mechanism is adopted for all methods to eliminate differences due to replay. PDQN is chosen as a representative parameterized action-space method. MPDQN is included because it uses branching ideas to reduce interference among action parameters. However, the two baselines differ from DPDQN in their architectural essence and degree of decoupling.

**Figure 9:**
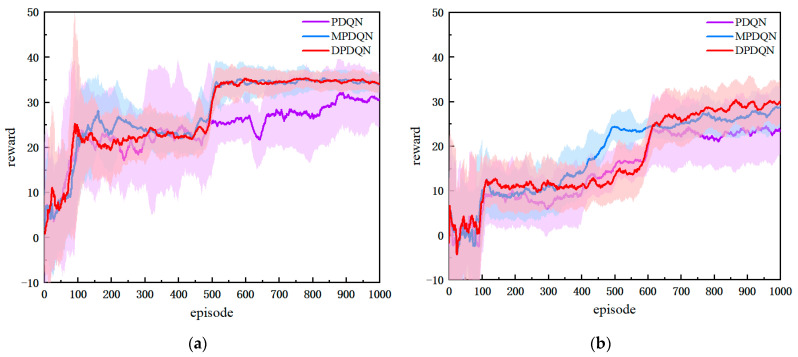
Comparison of Training Reward Curves Across Network Architectures: (**a**) Static Scenario; (**b**) Dynamic Scenario.

As shown in Figure 9, all three methods exhibit increasing cumulative rewards as training progresses, indicating gradual performance improvement. In the static environment, DPDQN converges faster and attains a stable average cumulative reward of approximately 36.36, higher than PDQN and slightly higher than MPDQN. Although DPDQN requires slightly more episodes to converge, its training is more stable, with a standard deviation of 2.07, which is lower than PDQN and comparable to MPDQN. This suggests reduced reward fluctuations and more stable policy convergence, largely due to effective semantic decoupling and suppressed interaction interference. In the dynamic environment, DPDQN maintains strong performance with an average reward of 34.71, outperforming PDQN and MPDQN. The average reward of DPDQN decreases by only 4.6 percent from static to dynamic settings, while PDQN and MPDQN decrease by 30.7 percent and 6.8 percent, respectively, indicating better performance and training stability. Table 3 summarizes convergence statistics.

The results in Table 4 further demonstrate the advantages of the multi-branch architecture in path-planning tasks. In static environments, all three algorithms achieve relatively high success rates. However, DPDQN exhibits a shorter average path length (25.27) and a higher average reward (35.75), indicating better efficiency and reward acquisition. In dynamic environments, the success rate of DPDQN reaches 0.83, which is significantly higher than that of PDQN and MPDQN. The path length and average reward of DPDQN are also superior to those of the baselines. This suggests that the multi-branch structure effectively reduces parameter interference between actions, ensuring that the strategy maintains high success rates and reward values in complex dynamic environments.

The performance gains arise because the multi-branch structure generates continuous parameters for each action independently, aligning control outputs with action semantics and enabling efficient path adjustment and obstacle avoidance under frequent environmental changes. The multi-branch design also reduces parameter interference among actions, improving control stability and preventing oscillations or failures during avoidance. In contrast, PDQN relies on a shared network, which can suffer from gradient interference in scenes with strong action semantics, limiting stability and parameter accuracy. MPDQN reduces dependence on irrelevant parameters through multiple forward passes, yet it remains a single-network design in essence, with branches only in the forward computation flow and fully shared underlying parameters. This makes it difficult to eliminate semantic coupling and interference completely. DPDQN, by design, achieves true multi-branch decoupling with an independent parameter sub-network for each discrete action, and output constraints (for example, Sigmoid for linear velocity and Tanh for angular velocity) that match action requirements. As a result, DPDQN avoids interaction gradient interference and preserves policy stability and interpretability in dynamic environments.

### 4.4. Analysis of Enhanced Empirical Mechanism Based on Transformer

To evaluate Transformer Enhanced Experience Replay (TER), we use a unified policy-gradient backbone and compare TER, uniform experience replay (ER), and prioritized experience replay (PER). All methods share the same state representation and policy architecture so that the replay mechanism is the only differing factor. This design isolates the impact of replay on sample utilization and policy optimization.

**Figure 10:**
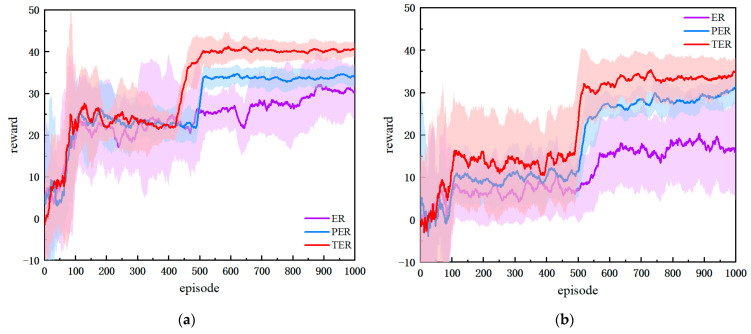
Comparison of Training Reward Curves Across Experience Replay Mechanisms: (**a**) Static Scenario; (**b**) Dynamic Scenario.

As shown in Figure 10, ER, PER, and TER all improve with training in both static and dynamic environments, but they differ in convergence speed, final performance, and stability. In the static environment, PDQN-TER rises rapidly and reaches a higher reward level after about 514 episodes, maintaining a sustained advantage thereafter. Its confidence band is narrower, indicating better policy quality and stability. PDQN-ER shows large fluctuations and lower stability. PDQN-PER converges faster than ER but exhibits substantial variance. These observations suggest that ER, which relies on random sampling, struggles to preserve causal chains in state transitions, leading to slow and unstable learning. PER improves the sampling of selected key experiences using TD error, but in tasks with long-range dependencies it may miss trajectory-level information and still converge slowly with high variance. PDQN-TER models entire trajectories globally, captures temporal dependencies among key states such as obstacle avoidance, turning points, and path replanning, and assigns higher sampling weights to such experiences. This improves learning efficiency and control accuracy while avoiding sequence-information loss in ER and sensitivity to noisy experiences in PER.

PDQN-TER adapts quickly to novel state distributions and maintains high rewards and stable convergence in dynamic obstacle environments, indicating strong environmental adaptability. In contrast, PDQN-PER can be misled by noisy experiences with high TD-error in dynamic scenes, which skews priority away from optimal trajectories and reduces generalization. ER lacks sequence modeling and struggles to adapt to environmental dynamics. By using self-attention to score trajectories with global semantic information, PDQN-TER increases the retention and sampling probability of the most informative state transitions, improving both training efficiency and task performance in complex scenes. Table 5 and Table 6 provide quantitative evidence.

These results show that DPDQN-TER emphasizes pivotal experiences in complex environments, improves policy stability and generalization, and achieves better path planning and higher task success rates in dynamic scenarios.

### 4.5. Cross-Environment Generalization Performance Verification

**Figure 11:**
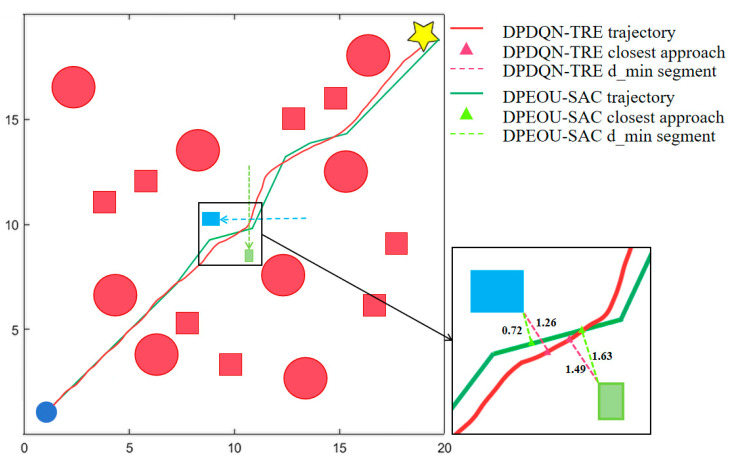
Trajectory Comparison of Different Algorithms in the Standardized Map Scenario.

To evaluate the generalization ability of the proposed algorithm across different environments, particularly its performance under non-customized map conditions, this study adopts path-planning scenarios published in previous research for replication experiments. In particular, we employ the scenario introduced in *“Unmanned Ground Vehicle Path Planning Based on Improved DRL Algorithm”* (Electronics, 2024) [39], as illustrated in Figure 11. This environment consists of a 20 × 20 grid map with static obstacles (depicted in red) and multidirectional dynamic obstacles (blue and green rectangles) moving at 2 m/s along the x and y axes, simulating complex dynamic conditions. The proposed DPDQN-TER algorithm is trained and tested on this map and compared with the baseline algorithm presented in the referenced study, in order to verify its policy transferability and generalization performance under cross-task and cross-environment conditions.

Figure 11 presents a comparison of trajectory diagrams in complex dynamic environments. The red trajectory corresponds to the DPDQN-TER method proposed in this paper, while the green trajectory represents the performance of DPEOU-SAC. Although DPEOU-SAC is able to avoid dynamic obstacle interference, DPDQN-TER reaches the target point more directly and efficiently, demonstrating superior adaptability to bidirectional moving obstacles. These results suggest that the proposed method ensures greater stability and reliability of path-planning performance when applied to novel environments.

**Figure 12:**
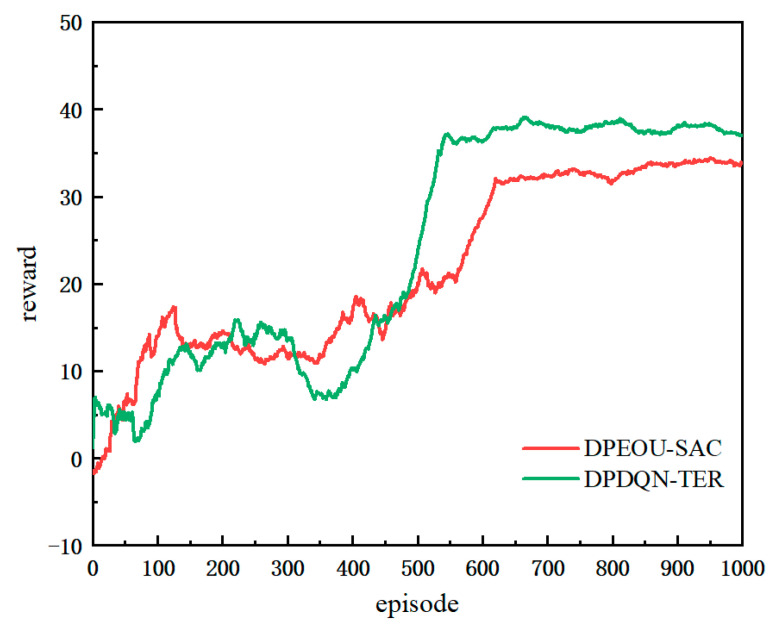
Training Curve Comparison of Different Algorithms in the Standardized Map Scenario.

Figure 12 illustrates the performance of the two algorithms in a reproducible environment, while Table 7 provides a detailed overview of the performance metrics used during testing. As shown in the results, the proposed DPDQN-TER algorithm achieves superior path-planning performance on the standard map, with a success rate of 0.96, shorter average path length, higher reward value, and faster convergence. Beyond success rate, path length, and reward, Table 7 also reports the minimum distance to obstacles. DPDQN-TER maintains larger minimum distances than the baseline in the standardized map, suggesting more conservative safety margins and fewer near-grazing events during navigation. These outcomes are clearly more effective than those of the DPEOU-SAC algorithm. This finding indicates that DPDQN-TER is effective not only in customized environments but also demonstrates strong generalization ability and performance under distribution shifts when applied to other task environments. The integration of Transformer-based experience modeling and a multi-branch policy structure plays a key role in improving policy stability and enhancing the recognition of critical states. In turn, this enables the policy to adapt to a wider variety of map distributions and dynamic task conditions.

## 5. Conclusions

In this study, we propose DPDQN-TER, a deep reinforcement learning–based method for autonomous navigation and path planning of mobile robots in dynamic environments. The method integrates a Transformer-based experience enhancement mechanism with a multi-branch parameter policy network. Its objective is to improve overall navigation performance (e.g., success rate and path length), training stability, and convergence efficiency in complex and dynamically changing obstacle environments. The proposed experience replay mechanism models the temporal and causal structure of experience sequences and identifies key state transitions—such as obstacle avoidance and turning points—through self-attention weights. Meanwhile, the multi-branch strategy structure enables decoupled control of different action semantics, thereby eliminating parameter interference between actions and improving the accuracy and response speed of continuous parameter generation. Experimental results from simulation environments show that the proposed algorithm consistently outperforms benchmark methods in both static and dynamic scenarios. Notably, the algorithm maintains a high success rate, shorter path lengths, and stable strategies, particularly in dynamic scenarios characterized by dense interference. This study therefore provides a feasible technical pathway for autonomous navigation of mobile robots in high-precision measurement tasks, such as large-scale structure fabrication.

The main advantages of the proposed approach are as follows:

1. Experience utilization with temporal perception: TER captures long-term state transition dependencies using Transformer modeling. It overcomes the limitations of traditional experience replay in preserving temporal structure and allows strategies to efficiently learn key decisions triggered by dynamic obstacles.
2. Decoupled action control architecture: DPDQN separates the shared parameter network into semantically independent sub-networks, eliminating gradient interference between actions and improving the accuracy of continuous parameter generation.
3. High-performance, stable adaptability in dynamic environments: The joint optimization framework enables mobile robots to maintain stable strategies under abrupt environmental changes, achieving a success rate of 92% in dynamic obstacle scenarios with only a 4.6% performance reduction compared with static environments.

Future research will focus on the following directions:

1. Lightweight temporal attention for experience modeling: On resource-constrained platforms, full Transformer modules can introduce non-trivial overhead. Future work will prioritize efficient attention variants (e.g., linear attention and state-space models) and hybrid compression strategies (e.g., pruning, quantization, and knowledge distillation) to reduce inference latency while preserving long-range temporal modeling capacity.
2. Adaptive branching mechanism: While the fixed multi-branch policy network is effective, greater flexibility may improve generalization across tasks. We will explore dynamic branching mechanisms in which the number and types of branches are selected or adapted online according to task context and environmental structure, potentially via lightweight architecture search or meta-policy selection.
3. Systematic Real-World Deployment and Robustness Benchmarking: We will extend validation beyond 2D simulation by migrating to physics-consistent 3D simulators with denser and faster dynamic obstacles, partial observability, and occlusions; calibrating sensor noise, dropout, and end-to-end latency, as well as actuation delay and saturation, from onboard logs and incorporating these effects through domain randomization, latency-aware state construction, and lightweight delay compensation; and conducting staged real-robot evaluation from hardware-in-the-loop to controlled indoor trials and small-scale field tests with safety envelopes and runtime distribution-shift monitors. Across these stages, we will report capacity-matched metrics (success rate, path length, and stability), deployment indicators (batch-1 latency and control frequency), computational footprint (parameter count and per-step FLOPs/MACs), and robustness under calibrated disturbances, thereby substantiating transfer of robustness to real-world deployments.

## Figures and Tables

**Table 1:** Network training parameter.

Parameters	Value
Learning rate	0.001
Batch Size	64
Replay memory size	10,000
Discount factor	0.99
Initial epsilon (Ɛ)	1
Max episodes	1000

**Table 2:** Performance evaluation indicators of different methods.

Method	Static Scenario			Dynamic Scenario			
	Success Rate	Path Length	Average Reward	Success Rate	Path Length	Average Reward	Minimum Distance
PDQN-ER	0.90	26.88	30.44	0.60	44.48	15.77	0.87
PDQN-TER	0.91	25.64	33.83	0.81	40.99	29.13	0.28
MPDQN-PER	0.93	23.80	39.73	0.83	39.67	23.82	0.59
DPDQN-TER	0.99	20.37	48.89	0.92	30.39	35.68	0.31

**Table 3:** Summary of Performance Metrics Across Network Architectures.

Method	Static Scenario			Dynamic Scenario		
	Reward Convergence Value	Convergence Round Count	Mean Standard Deviation	Reward Convergence Value	Convergence Round Count	Mean Standard Deviation
PDQN	26.51	569	2.18	18.35	651	6.33
MPDQN	32.71	518	1.99	33.67	508	4.63
DPDQN	36.36	528	2.07	34.71	653	5.00

**Table 4:** Comparison of Test Performance Across Different Network Architectures in Static and Dynamic Environments.

Method	Static Scenario			Dynamic Scenario		
	Success Rate	Path Length	Average Reward	Success Rate	Path Length	Average Reward
PDQN	0.90	26.88	30.44	0.60	44.48	15.77
MPDQN	0.87	26.4	33.11	0.72	38.36	29.33
DPDQN	0.88	25.27	35.75	0.83	34.68	33.66

**Table 5:** Summary of Performance Metrics Across Experience Replay Mechanisms.

Method	Static Scenario			Dynamic Scenario		
	Reward Convergence Value	Convergence Round Count	Mean Standard Deviation	Reward Convergence Value	Convergence Round Count	Mean Standard Deviation
PDQN-ER	26.51	569	2.18	18.35	651	6.33
PDQN-PER	34.08	485	2.44	30.46	583	3.83
PDQN-TER	40.55	514	1.78	34.28	519	3.59

**Table 6:** Performance Comparison of Different Experience Replay Mechanisms in Static and Dynamic Environments.

Method	Static Scenario			Dynamic Scenario		
	Success Rate	Path Length	Average Reward	Success Rate	Path Length	Average Reward
PDQN-ER	0.90	26.88	30.44	0.60	44.48	15.77
PDQN-PER	0.91	25.64	33.83	0.81	40.99	29.13
PDQN-TER	0.96	20.37	38.27	0.89	30.39	33.59

**Table 7:** Generalization Performance Comparison on the Map Proposed.

Method	Success Rate	Path Length	Average Reward	Minimum Distance
DPEOU-SAC	0.93	27.64	33.81	0.72
DPDQN-TER	0.96	25.36	38.74	1.26

## Data Availability

The original contributions presented in the study are included in the article; further inquiries can be directed to the author.
